# More than just appendicitis: incidental detection of a serrated polyp with malignant potential

**DOI:** 10.1093/jscr/rjaf1067

**Published:** 2026-01-16

**Authors:** Kamran Ahmad Malik, Nour Abu Asfour, Lutfi Ramadan Jarboa, Ahmad Zarour

**Affiliations:** Department of Acute Care Surgery, Hamad General Hospital, Hamad Medical Corporation, PO Box 3050, Doha, Qatar; Department of Pharmacy, Hamad General Hospital, Hamad Medical Corporation, PO Box 3050, Doha, Qatar; Department of Acute Care Surgery, Hamad General Hospital, Hamad Medical Corporation, PO Box 3050, Doha, Qatar; Department of Acute Care Surgery, Hamad General Hospital, Hamad Medical Corporation, PO Box 3050, Doha, Qatar

**Keywords:** polyp, acute appendicitis, appendectomy, case report

## Abstract

Acute appendicitis is a common surgical emergency, sometimes revealing incidental neoplastic lesions such as serrated polyps, which have malignant potential through the serrated pathway of colorectal carcinogenesis. A 41-year-old healthy female presented with one-day abdominal pain, initially periumbilical then localized to the right lower quadrant, with nausea and vomiting. Imaging confirmed acute appendicitis, and laparoscopic appendectomy was performed. Histopathology showed acute inflammation and an incidental serrated polyp confined to the mucosa, without dysplasia or malignancy. The patient recovered uneventfully and was referred for colonoscopy to assess for synchronous colorectal lesions. Routine histopathological evaluation of appendectomy specimens is essential, especially in patients over 40. Incidental serrated polyps, though rare, require further investigation due to their association with colorectal neoplasia. Early detection enables appropriate surveillance and may contribute to colorectal cancer prevention.

## Introduction

Acute appendicitis is a common surgical emergency, usually caused by luminal obstruction from fecaliths, lymphoid hyperplasia, or less commonly, neoplasms like appendiceal polyps [1]. Epithelial neoplasms appear in 0.2%–0.3% of appendectomy specimens, some exhibiting serrated histology [2].

Serrated polyps—including hyperplastic polyps, sessile serrated lesions, and traditional serrated adenomas—are rare in the appendix [3] but important due to their role in the serrated pathway of colorectal carcinogenesis. These polyps are typically incidental findings on histopathology, as they are asymptomatic and rarely identified during surgery [4–6].

The coexistence of acute appendicitis and serrated polyps is uncommon and not well understood; however, patients over 40 presenting with appendicitis should be evaluated for possible neoplasia [7].

Nonoperative management (NOM) of uncomplicated appendicitis is increasingly favoured [8, 9] but eliminates histop-athological examination, risking missed incidental lesions. Surgical resection remains vital both for treatment and diagnosis.

This case underscores the importance of histopathological evaluation to detect rare but clinically significant lesions such as serrated polyps and highlights the need for appropriate postoperative surveillance.

## Case presentation

A 41-year-old previously healthy woman presented with a 1-day history of abdominal pain that started periumbilically and localized to the right lower quadrant, accompanied by nausea and three episodes of non-bloody vomiting. She denied fever, anorexia, urinary symptoms, or bowel changes. She reported a similar and self-resolving episode, months earlier. Her surgical history included a caesarean section and myomectomy; she had no chronic illnesses or medications.

**Figure 1:**
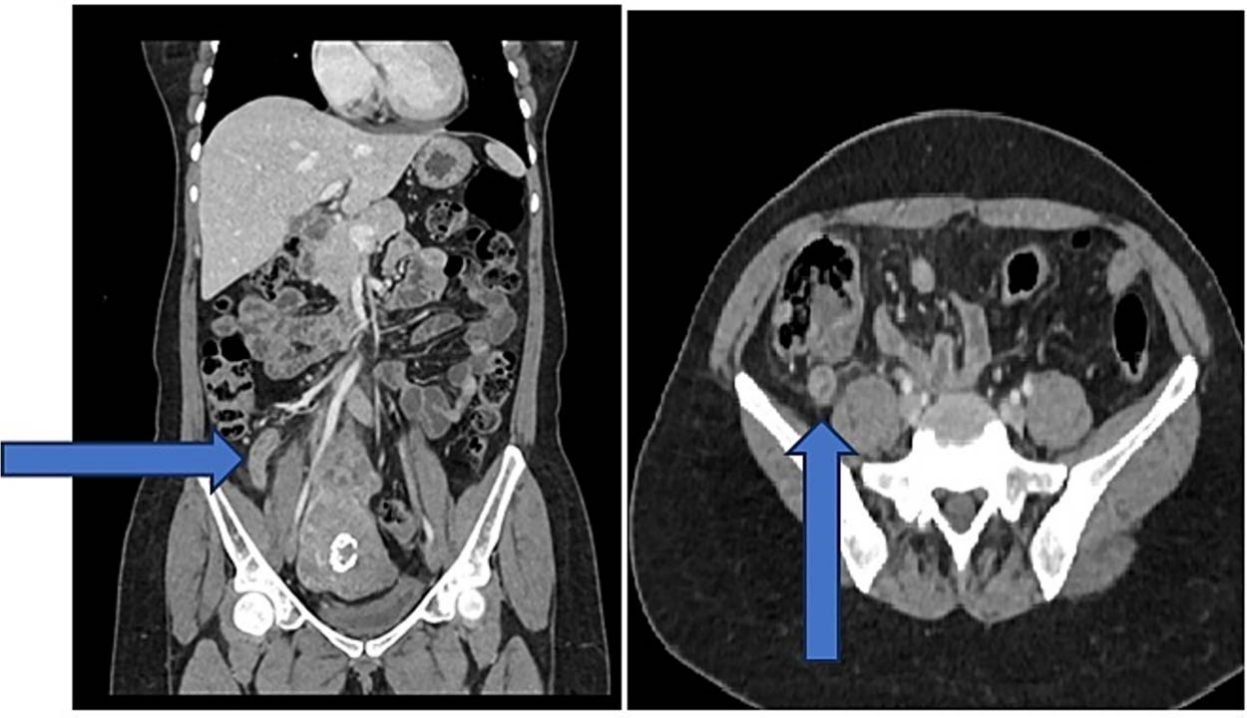
CT images with blue arrow pointing to inflamed appendix.

On examination, she was afebrile and stable, with localized right iliac fossa tenderness, rebound tenderness, mild epigastric tenderness, and a positive Rovsing’s sign. Labs showed a white blood cell count of 10.4 × 10^9^/L and C-reactive protein of 45 mg/L. Contrast-enhanced computed tomography (CT) revealed an 11 mm dilated appendix with mural enhancement, minimal fat stranding, slight free fluid, and small mesenteric lymph nodes, consistent with uncomplicated acute appendicitis (Fig 1).

**Figure 2:**
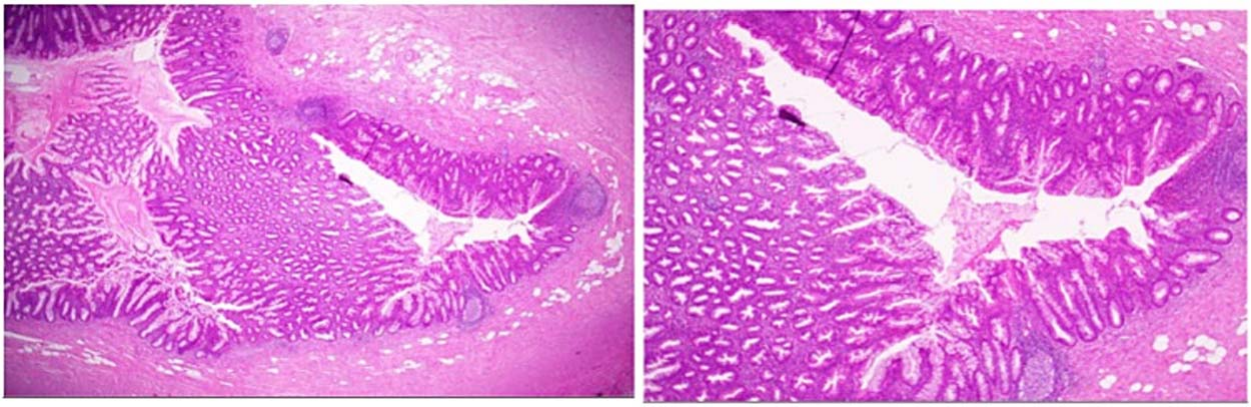
Low-power hematoxylin and eosin-stained sections of the appendix (4× and 10× magnification) demonstrating prominent crypt serration within the appendiceal mucosa with the characteristic saw-toothed crypt architecture and increased mucin production.

**Figure 3:**
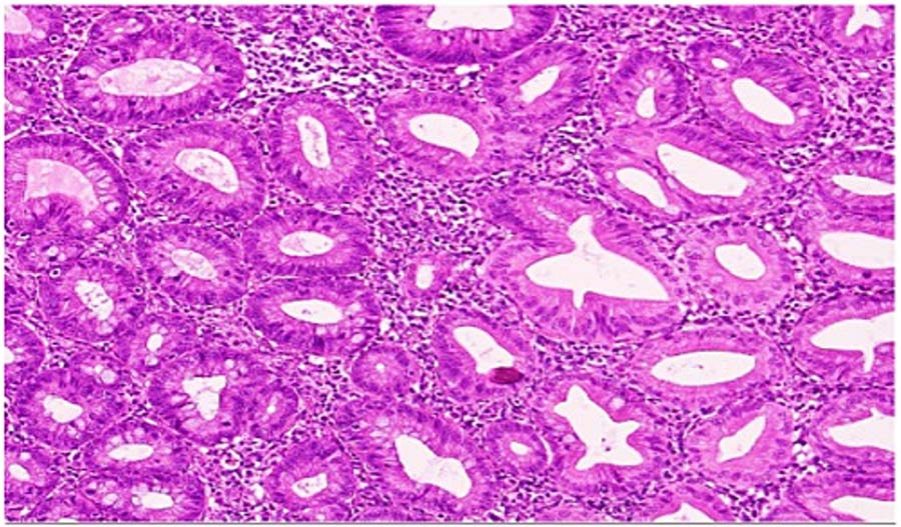
High-power view showing comparison of the adjacent normal appendiceal mucosa with unremarkable straight crypts lining the lumen and the serrated architecture of the polyp.

**Figure 4:**
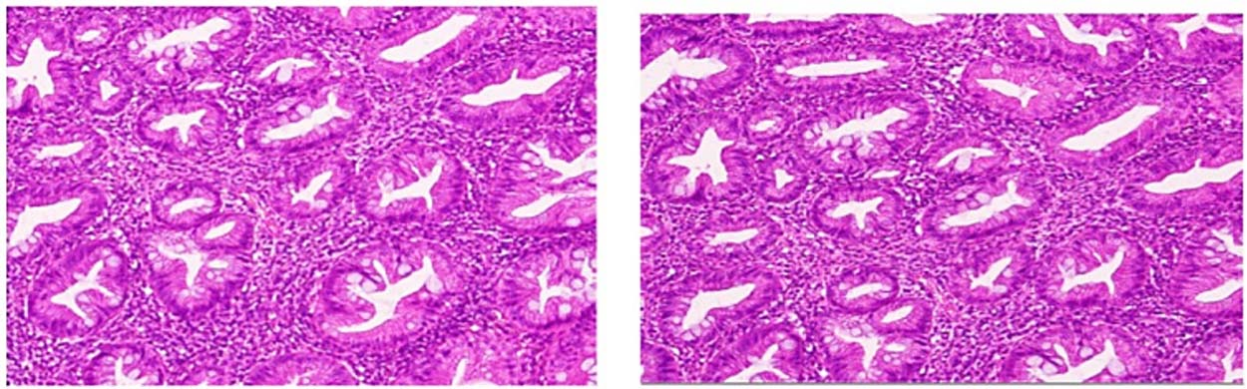
High-power magnification highlighting the serrated configuration of the crypt epithelium in detail, with luminal tufting and goblet cell-rich lining.

She underwent an uncomplicated laparoscopic appendectomy. Histopathology confirmed acute appendicitis and incidentally identified a serrated polyp confined to the mucosa without dysplasia or malignancy (Figs. 2—4). Postoperative recovery was uneventful; she was discharged stable and asymptomatic at 2-week follow-up. Given the incidental finding, colonoscopic evaluation was arranged to assess for synchronous colorectal lesions.

## Discussion

Appendiceal neoplasms are rare, with a reported incidence of 0.2%–3.2% in appendectomy specimens [4]. These include low-grade appendiceal mucinous neoplasms, neuroendocrine tumours, and serrated polyps. Though uncommon, serrated polyps are premalignant and represent part of the serrated pathway of colorectal tumorigenesis [4].

The incidental discovery of serrated polyps—as in our case—demonstrates the diagnostic value of routine histopathological examination, even in cases of uncomplicated appendicitis.

Several studies have evaluated the association between appendicitis and colorectal malignancy. For example, Mohamed *et al.* found that among patients aged ≥55 years undergoing appendectomy for acute appendicitis, 1.6% were later diagnosed with caecal carcinoma [7]. Another study reported a 0.85% incidence of colon cancer post-appendectomy, with a median diagnosis interval of 5.8 months [10]. These findings support colonoscopic evaluation in older patients presenting with appendicitis.

However, the growing trend toward NOM for uncomplicated appendicitis introduces a potential diagnostic pitfall. While trials such as APPAC support antibiotic therapy as an alternative to surgery [11], NOM inherently omits histopathological assessment. Our case illustrates the clinical value of surgery in detecting incidental lesions with malignant potential.

At present, no standardized surveillance guidelines exist for patients with incidentally discovered appendiceal serrated polyps. The decision to pursue colonoscopy is often left to the clinician’s discretion. However, due to the possibility of synchronous or metachronous lesions, particularly with serrated histology, further research is warranted to guide evidence-based follow-up protocols.

This case adds to the limited literature on the coexistence of acute appendicitis and serrated polyps. It reinforces the role of surgery not only as treatment but also as an opportunity for early detection of premalignant lesions.

### Limitations

This is a single-case report, limiting generalizability. In addition, long-term follow-up including colonoscopic findings is pending, which restricts assessment of the broader implications.

## Conclusion

This case emphasizes the importance of routine histopathological examination of appendectomy specimens, particularly in patients over 40 years of age. The incidental detection of a serrated polyp highlights the potential for underlying or synchronous colorectal neoplasia. Further research is needed to determine the true prevalence of these lesions in appendicitis and to develop standardized guidelines for follow-up and risk stratification. Surgical management not only addresses the acute condition but also provides a crucial opportunity for early detection and prevention of colorectal cancer.
